# *Bacillus amyloliquefaciens* SQ-2 and Biochar: A Promising Combination for Enhancing Rice Growth in Pb/Al-Contaminated Acidic Soils

**DOI:** 10.3390/microorganisms13071556

**Published:** 2025-07-02

**Authors:** Guohui Gao, Xue Li, Jiajun Ma, Yumeng Cui, Ming Ying, Lei Huang, Meitong Li

**Affiliations:** Tianjin Key Laboratory of Organic Solar Cells and Photochemical Conversion, College of Chemistry and Chemical Engineering, Tianjin University of Technology, Tianjin 300384, China; gaoguohui@stud.tjut.edu.cn (G.G.); huaxuehuagong12@126.com (X.L.); martintin@stud.tjut.edu.cn (J.M.); 15127176578@163.com (Y.C.); ym@tjut.edu.cn (M.Y.)

**Keywords:** *Bacillus amyloliquefaciens*, biochar, metal, PGPR

## Abstract

In this study, *Bacillus amyloliquefaciens* SQ-2, previously isolated from a commercial watercress paste, was investigated for its potential in promoting rice growth in Pb/Al-contaminated acidic soil, especially when used in conjunction with corn straw biochar. Firstly, the physiological properties of rice were enhanced, with the activities of catalase and superoxide dismutase increasing by 162.5% and 162.9%, respectively. Additionally, the total phenolic and chlorophyll contents of rice increased by 17.6% and 83.7%, respectively. Secondly, the nutrient content of the rice rhizosphere soil was improved. In particular, nitrate nitrogen, available potassium, and sucrase were enhanced by 9.4%, 45.9%, and 466.8%, respectively. Moreover, SQ-2–biochar was demonstrated to have a notable capacity for removing Pb^2+^ and Al^3+^. The mineralization of Pb^2+^ and Al^3+^ was achieved through the use of SQ-2–biochar, as revealed by SEM-EDS, XRD, XPS, and FT-IR analyses, with the main precipitates being Pb_3_(PO_4_)_2_ and AlPO_4_. Functional groups such as C-O-C, C=O, N-H, P-O, and -O-H on the microbial surface were found to be involved in the biosorption process of Pb^2+^ and Al^3+^. In summary, SQ-2–biochar can effectively mineralize Pb^2+^ and Al^3+^, enhance the physiological properties of rice, and improve soil nutrients, thereby augmenting the antioxidant capacity, photosynthesis, and stress resistance of rice and ultimately promoting rice growth.

## 1. Introduction

Rice is a staple food for a large portion of the global population, particularly in China. However, in recent years, the increasing contamination of rice by metals has become a significant concern [1]. Metals, especially in the roots, can induce oxidative stress, leading to damage to plant cells and thereby adversely affecting rice growth [2]. Globally, approximately 30% of land area and over 50% of potential arable land are acidic soils [3]. The presence of metals in such soils has been shown to have a deleterious effect on rice, resulting in impaired nutrient absorption, stunted growth, reduced transpiration, and impaired photosynthesis. The relatively low pH value of acidic soil (<5.5 or 6) is often associated with the accumulation of aluminum (Al) [4]. In soil, Al is commonly found in the form of insoluble aluminosilicates or alumina. However, under acidic conditions, it transforms into exchangeable Al, which impedes the absorption and utilization efficiency of nitrogen by roots during growth [4]. Additionally, lead (Pb) is one of the prominent pollutants in soil worldwide. High levels of Pb can result in the poor growth of rice and have adverse effects on the absorption of macronutrients and plant chlorophyll content [5]. In China, the percentage of Pb pollution in soil has reached 1.5% [6], emphasizing the need for urgent mitigation measures to address this pressing environmental concern.

The application of Plant Growth-Promoting Rhizobacteria (PGPR) has been demonstrated to enhance plant growth and enhancing plant resistance to diseases. To date, numerous PGPR isolated from diverse environments have been demonstrated to improve crop productivity. For example, inoculation with *Priestia* sp. LWS1 increased rice yield by 19 percent [7]. The mechanisms by which PGPR enhance plant growth include the following: enhanced material cycling, facilitated nutrient absorption, the regulation of plant hormone synthesis, and the alleviation of environmental stress [8]. PGPR can directly promote plant growth through processes such as nitrogen fixation, phosphorus solubilization, potassium solubilization, and the promotion of hormone production required by plants. Indirectly, they can produce antagonistic compounds like enzymes, iron carriers, and antibiotics [9]. Moreover, PGPR can adsorb metals, reducing their toxicity and facilitating plant growth. It has been found that many soil microorganisms, including PGPR, possess detoxification mechanisms for metals. These mechanisms involve actively releasing metals, converting metals to less toxic valence states, adsorbing and reducing certain metal ions, and secreting polysaccharides to bind metals [10].

Currently, PGPR inoculants are widely utilized in agricultural fertilizers, soil improvement, and bioremediation. These bacteria have the capacity to influence the composition of rhizosphere communities, thereby enhancing soil fertility and providing a conducive environment for plant rhizosphere communities. The efficacy of PGPR in enhancing crop yield varies depending on the specific crop type, with some studies reporting an increase of up to 57% [11]. In addition to strengthening plants’ defenses against specific pathogens, PGPR have been shown to assist plants in tolerating certain abiotic stresses [12]. Among the various bacteria that have been the focus of study, *Bacilli* and *Bacilli*-like bacteria are the most extensively researched [13]. These bacteria are ubiquitous in soil and have been isolated from the rhizosphere of many different plants. Numerous studies have reported their beneficial effects on plants and their ability to control soil-borne diseases [14]. Microbial fertilizers based on *Bacillus* species, such as *Bacillus megaterium*, *Bacillus subtilis*, *Bacillus licheniformis*, and *Bacillus sphaericus*, are widely used in agriculture, with *Bacillus sphaericus* accounting for approximately half of the commercially available bacterial biocontrol agents [15].

Corn stover is a cost-effective and readily available material compared to manure, and its pyrolysis into biochar at high temperatures can lead to the reuse of agricultural waste. In addition, straw biochar is rich in potassium, which can be applied to the soil to improve plant resistance. Therefore, we chose corn stover as the raw material for biochar.

Straw biochar, a carbon-based solid obtained from biomass pyrolysis, contains complex organic matter, a large amount of carbohydrates, inorganic substances, and other components, making it a valuable source of nutrients for plant growth [16]. Corn stover is a cost-effective and readily available material compared to manure, and its pyrolysis into biochar at high temperatures can lead to the reuse of agricultural waste. In addition, straw biochar is rich in potassium, which can be applied to the soil to improve plant resistance [17]. Therefore, we chose corn stover as the raw material for biochar. When added to soil, crushed biochar can increase soil porosity, making the soil more loose [18]. Biochar is alkaline and can effectively raise the pH of acidic soil when applied [19]. The combination of biochar and functional bacterial or fungal strains is regarded as an effective and emerging strategy for the sustainable remediation of polluted soil. Some reviews [20,21] have proposed potential interaction mechanisms between biochar and soil microorganisms. Biochar’s porous structure can provide shelter for microorganisms, supply nutrients, and change the properties of the soil, soil enzyme activity, and rhizosphere microbial communities. It has been demonstrated that the process can enhance the conversion and degradation of pollutants through the mechanisms of adsorption and electron transfer [22]. However, the binding mechanism between biochar and exogenous functional microorganisms remains to be elucidated, and the underlying mechanism of the combined biochar–PGPR remediation of soil metal pollution has not been fully elucidated [23]. 

The combined application of biochar and PGPR is effective in promoting plant growth and reducing heavy metal content in plants and soil. Zhao [20] et al. reported that the combination of biochar and PGPR significantly reduced Pb toxicity and increased plant biomass. However, there are currently few resources of phytoproducing bacteria that can be used for rice production in acidic soils because the activities of key enzymes in most of them are reduced under acidic conditions, and the solubility of heavy metals is increased under acidic conditions, which further exacerbates the oxidative stress of the microorganisms. Therefore, it is crucial to screen for strains that can enhance rice production under acidic conditions. SQ-2 was pre-screened in the laboratory for acid- and heavy metal-tolerant strains. This study was conducted with the objective of investigating the combined effect of a growth-promoting bacterium (SQ-2) and straw biochar on rice growth in acid soil contaminated with Al^3+^ and Pb^2+^. The objective of this research is to establish a theoretical framework for understanding the adsorption mechanism of the strain–biochar combination and to develop a model that can account for the endogenous mechanism in rhizosphere soil.

## 2. Materials and Methods

### 2.1. Strain, Medium, Soil, and Biochar

*Bacillus amyloliquefaciens* SQ-2 was previously isolated from commercial watercress paste in our laboratory. Specifically speaking, the watercress paste was serially diluted in sterile water before 20 μL samples were spread onto Luria–Bertani (LB) agar medium. These samples were then incubated at 25 °C for 48 h. After incubation, bacterial colonies were purified by multiple streaking on LB agar plates. And SQ-2 has been proven to demonstrate excellent antibacterial properties [24,25]. All the medium formulations were liquid media, and 20 g/L agar powder was added to prepare solid media. The pH of the media was adjusted to a range of 7.0–7.2 using a sodium hydroxide solution and sterilized at 121 °C for 30 min. Sucrose and dextrose were separately sterilized and then added to the media in portions. The metal ion solution was subjected to filtration and sterilization using a filter prior to its incorporation into the culture medium.

Luria–Bertani (LB) medium (g/L): peptone 10 g, yeast extract powder 5.0 g, NaCl 10 g; potassium-solubilizing medium (g/L): sucrose 5 g, dextrose 5 g, yeast extract powder 0.5 g, (NH_4_)_2_SO_4_ 0.5 g, MgSO_4_·7H_2_O 0.3 g, Na_2_HPO_4_ 2 g, FeSO_4_·7H_2_O 0.02 g, MnSO_4_·7H_2_O 0.03 g, potassium feldspar 4 g; Pikovskaya (PKO) medium (g/L): Ca_3_(PO_4_)_2_ 5 g, dextrose 10 g, (NH_4_)_2_SO_4_ 0.5 g, NaCl 0.2 g, MgSO_4_·7H_2_O 0.1 g, KCl 0.2 g, yeast extract powder 0.5 g, MnSO_4_·7H_2_O 0.03 g, FeSO_4_·7H_2_O 0.03 g.

The soil was collected from the Mingli Farm of Tianjin University of Technology. The basic physical and chemical properties were as follows: a pH of 5.83, total phosphorus at 446 mg/kg, total nitrogen at 521 mg/kg, total potassium at 1.03%, available phosphorus at 18.39 mg/kg, available nitrogen at 16.33 mg/kg, and available potassium at 62.52 mg/kg.

The biochar used in this study was derived from corn stover, with the following basic physical and chemical properties: organic carbon at 42.21%, total nitrogen at 0.74%, total phosphorus at 2.31%, total potassium at 16.12%, mineral content at 7.23%, and a pH of 9.46.

### 2.2. Determination of Growth-Promoting Characteristics of SQ-2

#### 2.2.1. Detection of Phosphorus Solubilization Ability of SQ-2

A single colony of SQ-2 was selected and cultured in LB medium for 3 days. After centrifugation, a bacterial suspension with an OD_600_ value of 1 was prepared using sterile water and inoculated into the PKO medium at an inoculation rate of 2%. The culture was incubated on a shaker at 25 °C and 120 r/min. After 1 day of cultivation, the culture medium was centrifuged to obtain the supernatant, which was filtered through phosphate-free filter paper. Phosphorus solubility was determined using the molybdenum blue colorimetric method [26], and the pH value of the medium was measured using a pH meter.

#### 2.2.2. Detection of Potassium-Solubilizing Ability of SQ-2

The strain was incubated in the potassium-solubilizing medium at 25 °C and 120 r/min for 8 days. The precipitate was retained by centrifugation at 10,000 rpm for 10 min and then dried in an oven for 12 h. The surface morphology of the potassium feldspar was observed using SEM.

### 2.3. Rice Soil Culture Experiment

High-temperature carbonized corn straw biochar was sieved through a 100-mesh sieve, and approximately 1.5 g of biochar was added to each pot containing 400 g of soil. Acidic soil with a pH of around 5.8 was selected, and Al_2_(SO_4_)_3_·18H_2_O and PbC_4_H_6_O_4_·3H_2_O were used to prepare the mother liquor. The mother liquor was added to the control group (M group), the SQ-2 group (S + M group), and the SQ-2–biochar group (S + M + C group). All soils with metals were aged in a cool place for 2 months. The concentrations of total lead and total aluminum after soil aging were 380.42 ± 23.15 mg/kg and 365.12 ± 18.12 mg/kg, respectively. The experiment was conducted in a growth chamber under the following environmental conditions: daytime temperature was maintained at 28 °C, nighttime temperature at 25 °C, and humidity at 65%, and a photoperiod of 12 h of light and 12 h of dark was used. Deionized water was added daily to keep the water level about 3 cm above the soil surface during growth. On the 2nd and 10th day of the experiment, 25 mL of a 1 × 10^9^ CFU/mL bacterial suspension was injected into the rhizosphere soil of the plants using a syringe. From the 20th to the 25th day of the experiment, plant leaves were collected for the detection of chlorophyll, enzyme activity, and substances such as malondialdehyde. Three different points were taken from each flowerpot, and the soil was mixed to obtain a total of 10 g. The soil was air-dried and sieved to detect the available nitrogen, phosphorus, and potassium in the soil, while some fresh soil was retained for the detection of sucrase.

Chlorophyll content was determined by the direct acetone–ethanol extraction method [27]. The activities of catalase (CAT) and peroxidase (POD) were quantified using the UV-absorbance and guaiacol methods, respectively [28]. Sucrase, total phenols, malondialdehyde, soil available phosphorus, available potassium, ammonium nitrogen, and nitrate nitrogen were detected using commercial testing kits from Hefei Laier Biotechnology Co., Ltd. (Hefei, China).

### 2.4. Characterization of SQ-2–Biochar Adsorption of Pb^2+^ and Al^3+^

LB media containing 100 mg/L of Pb^2+^ and 100 mg/L of Al^3+^ were prepared, and strain SQ-2 was inoculated into the metal-containing media at a 2% inoculation rate. The culture was shaken in a shaker at a constant temperature of 25 °C and 120 r/min. After 2 days, the bacterial cells were retained by centrifugation at a gradient of 1000 and 10,000 rpm for 10 min, washed repeatedly with sterile water, and centrifuged again three times. Finally, the bacterial cells were concentrated in a 1.5 mL centrifuge tube. A total of 1 mL of 2.5% glutaraldehyde solution was transferred into the centrifuge tube, and the sample was left to stand for 1 h in a ventilated area. The samples were dehydrated with 50%, 70%, 90%, and 95% ethanol for 15 min each and finally dehydrated with anhydrous ethanol for 15 min. After drying in a drying oven at 60 °C for 24 h, the prepared sample was observed under a field emission scanning electron microscope.

The bacterial cells were freeze-dried using a freeze-dryer, ground after drying, and sieved through a 60-mesh screen. The crystal structure of the samples was determined using an X-ray diffractometer (XRD, Rigaku Ultima, Tokyo, Japan) at a scattering angle of 2θ = 5–50° and a scanning rate of 30°/min. X-ray photoelectron spectroscopy (XPS, ESCALAB250Xi, Thermo Scientific, Waltham, MA, USA) was used to determine the metallic elements on the surface of the samples and the morphology of their presence. The sample powder and potassium bromide, at a ratio of 1:200, were pressed together to form tablets, and the changes in the functional groups of the bacteria after the adsorption of heavy metals were determined using a Fourier Transform Infrared Spectrometer (FTIR, STA6000-TL9000-Clarus SQ8, Bio-Rad Inc., Hercules, CA, USA) in the wavelength range from 400 to 4000 cm^−1^.

### 2.5. Transcriptome Analysis of SQ-2 Adsorbed Pb^2+^ and Al^3+^

A corresponding amount of metal solution was filtered and added dropwise to 250 mL of LB liquid culture medium to obtain 100 mg/L Pb^2+^ and Al^3+^. The seed solution of SQ-2 was cultured in LB medium to an OD_600_ value of 1.6–1.8 and then added to the above two metal media at a 2% inoculation amount. The strain SQ-2 culture medium without added metals was used for the control group. After 36 h, the bacterial cells were collected by centrifugation under sterile conditions and rinsed with sterile water. The prepared bacterial cells were frozen in liquid nitrogen and stored in a −80 °C refrigerator. The sample was placed in dry ice and sent to Majorbio pharmaceutical Company (Shanghai, China) for testing.

Complete RNA was extracted from the tissue samples, and then the concentration and purity of RNA were detected. The RIN value was determined using Agilent 2000. The total amount of RNA required for a single library construction was 2 μg with a concentration of ≥100 ng/μL and an OD_260_/_280_ between 1.8 and 2.2. After removing rRNA and adding fragmentation buffer, the intact long mRNA was randomly interrupted. The synthetic cDNA was reverse-transcribed, and the double-stranded cDNA structure was made into a sticky end. End Repair Mix was added to make it a flat end, and then an A base was added to the 3’ end to connect the Y-shaped joint. After digesting the second strand of cDNA with UNG enzyme, 2 × 150 bp/300 bp sequencing was performed using the sequencing platform.

Expression quantitative software such as RSEM, Kallisto, and Salmon was used to analyze gene expression. The raw counts among samples were standardized based on the TMM method, and the differential expression analysis between groups was performed using DEGseq (1.48.0) software. Using non-parametric tests for statistical analysis. Enrichment analysis of GO and KEGG pathways was also carried out.

### 2.6. Data Analysis

The differences in various indicators between different treatments were analyzed using the Duncan method. Pearson’s correlation analysis was used to analyze the correlation between soil nutrient composition, soil enzyme activity, and pH after inoculation with *Bacillus amyloliquefaciens* SQ-2. Excel 2016 and SPSS 25.0 were used for data analysis, and Origin 2021 and Adobe Photoshop 2021 were used for mapping.

## 3. Results

### 3.1. Phosphorus/Potassium-Solubilizing Capability of B. amyloliquefaciens SQ-2

**Figure 1:**
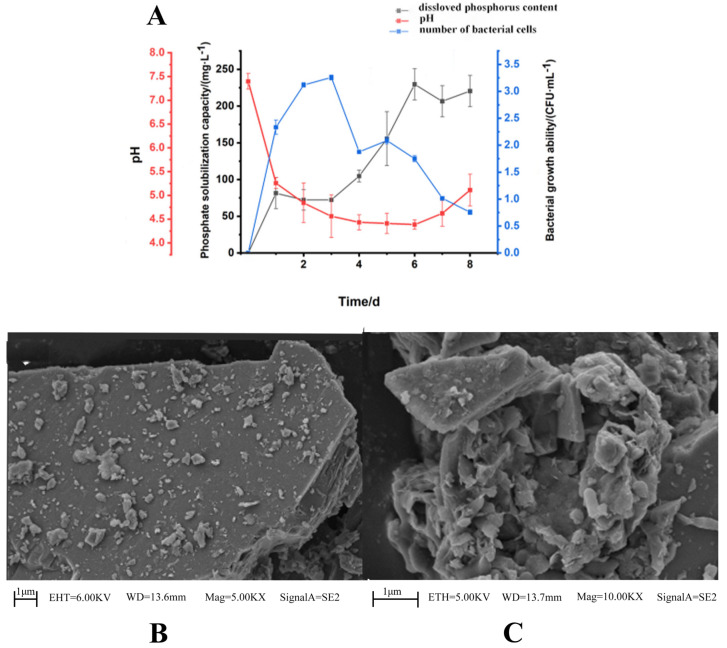
Phosphorus/potassium-solubilizing capability of SQ-2. (**A**): determination of phosphorus solubility of SQ-2; (**B**): SEM image of potassium feldspar; (**C**): determination of potassium solubility of SQ-2.

PGPR can directly promote plant growth by dissolving phosphorus and potassium. The solubility of phosphorus is usually directly related to the acid production capacity of the microorganisms. Therefore, changes in the pH value of the culture can reflect the level of phosphorus solubility [29]. As depicted in Figure 1A, after 1 day of incubation, the pH of the culture medium dropped rapidly to 5.3. Subsequently, with the progression of incubation time, the pH continued to decline. During the first two days of incubation, the strain exhibited its maximum growth rate, which coincided with a sharp decrease in the pH of the medium. As the pH diminished, the growth of the strain also began to wane. Remarkably, on the sixth day, the dissolved phosphorus content in the culture medium reached 245 mg/L, providing further evidence of SQ-2’s proficient phosphorus-solubilizing ability.

Soil mineral potassium constitutes 92% to 98% of the total potassium and is typically unavailable for plant uptake [30]. Hence, potassium feldspar was utilized to examine the potassium-dissolving capacity of SQ-2. Figure 1B presents an SEM image of potassium feldspar powder. The surface of the potassium feldspar was observed to be relatively flat and had several distinct angles, with a three-dimensional morphology. Figure 1C showcases an SEM image of the strain-potassium feldspar co-culture. The surface of potassium feldspar became rough due to corrosion, and the edges were no longer visible. The comparison shows that SQ-2 has a certain degree of potassium-dissolving ability.

### 3.2. SQ-2–Biochar Promotes Rice Growth in Pb/Al Acidic Soil

In accordance with the findings of previous research, the combined application of biochar and PGPR has been demonstrated to effectively enhance plant growth [31]. Therefore, we incorporated SQ-2–biochar into potted rice experiments to assess its impact on rice growth in Pb/Al-contaminated acidic soil. As presented in Table 1, both the SQ-2 group (S + M) and the SQ-2–biochar group (S + M + C) significantly enhanced the dry and fresh weights of rice stems. The stem fresh weights were 0.43 g and 0.44 g, respectively, representing increases of 38.7% and 41.9% compared to those in the Al/Pb group (M). The stem dry weight in both groups was 0.04 g, which was 33.3% higher than that of the Al/Pb group (M). Furthermore, an enhancement in plant height was observed, with values of 11.53 cm and 11.73 cm recorded in the SQ-2 and SQ-2–biochar groups, respectively, representing increments of 19.2% and 21.3% relative to the Al/Pb group (M). Notably, the fresh weight restoration of roots was only significantly effective in the SQ-2–biochar group (S + M + C), whereas the repair effects on the dry weight of roots and stem thickness were not statistically significant. Figure S1 illustrates the growth status of rice in the seedling stage in the four soil treatments.

### 3.3. Mechanisms of SQ-2–Biochar in Promoting Rice Growth in Pb/Al Acidic Soil

#### 3.3.1. The Effects of SQ-2–Biochar on the Physiological Properties of Rice in Pb/Al Acidic Soil

**Figure 2:**
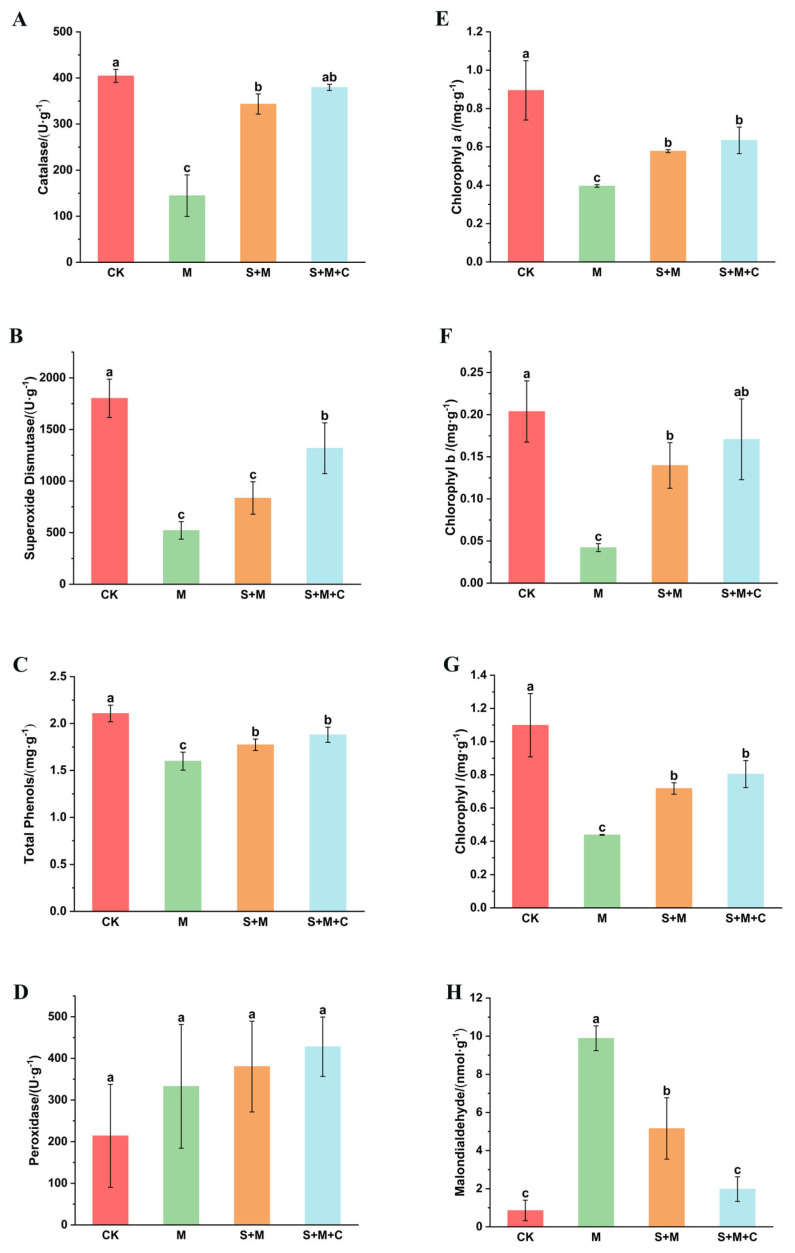
The effects of SQ-2–biochar on the physiological properties of rice in Pb/Al soil. (**A**): The activity of catalase. (**B**): The activity of superoxide dismutase. (**C**): Total phenol content in the plants. (**D**): The activity of peroxidase. (**E**): The content of chlorophyll a. (**F**): The content of chlorophyll b. (**G**): The content of total chlorophyll. (**H**): The content of malondialdehyde. Different letters in the graphs indicate significant differences at the *p* < 0.05 level.

As shown in Figure 2, in the four soil treatments, the catalase, superoxide dismutase, total plant phenol, and chlorophyll contents in the rice of the SQ-2 group (S + M) and SQ-2–biochar group (S + M + C) were significantly elevated compared to those in the Al/Pb group (M) (*p* < 0.05). Specifically, the catalase levels in the SQ-2 group and SQ-2–biochar group increased by 137.5% and 162.5%, respectively (Figure 2A). Concurrently, superoxide dismutase activity exhibited a 60.14% and 162.9% increase, respectively (Figure 2B). The total phenolic content of rice rose by 10.9% and 17.6%, respectively (Figure 2C). Concurrently, the total chlorophyll content exhibited a marked increase of 63.8% and 83.7%, as depicted in Figure 2G. Despite the observed variations in peroxidase content among the four experimental groups, these changes did not attain statistical significance (Figure 2D).

Under adverse conditions, malondialdehyde often undergoes membrane lipid peroxidation, leading to protein cross-linking polymerization and cytotoxicity [32]. In our study, the content of malondialdehyde in the SQ-2 group and SQ-2–biochar group was significantly reduced compared to that in the Al/Pb group (M), with decreases of 47.8% and 80%, respectively (Figure 2H).

#### 3.3.2. The Effects of SQ-2–Biochar on the Rhizosphere Soil Nutrients of Rice in Pb/Al Acidic Soil

**Figure 3:**
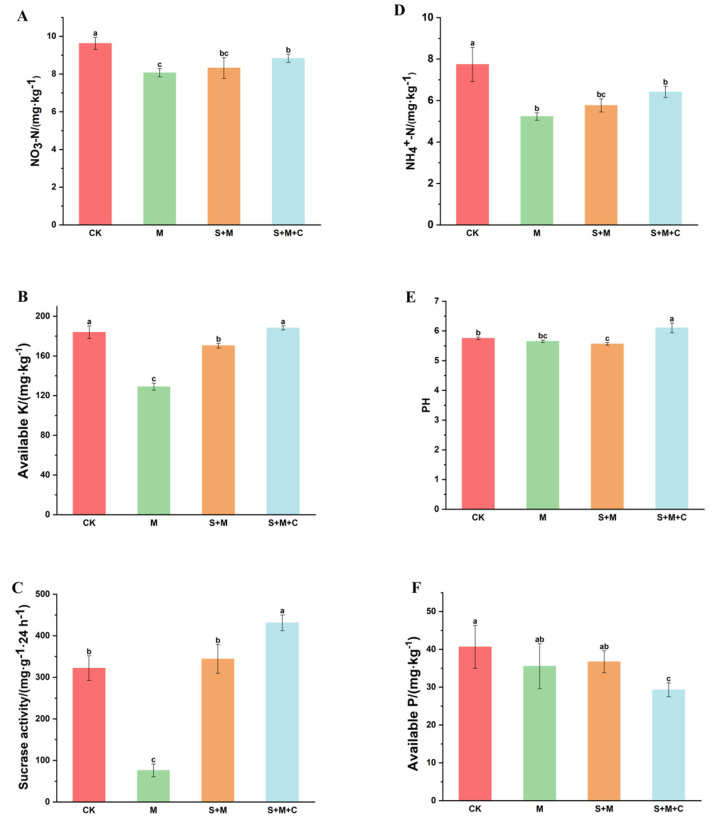
Efects of SQ-2–biochar on rhizosphere soil nutrients of rice in Pb/Al soil. (**A**): Content of nitrate nitrogen. (**B**): Content of available K. (**C**): Sucrase activity. (**D**): Content of ammonium nitrogen. (**E**): pH value. (**F**): Content of available P. Different letters in the graphs indicate significant differences at the *p* < 0.05 level.

The combined application of biochar and PGPR has been demonstrated to enhance soil nutrients, thereby promoting plant growth and increasing yield [33]. As illustrated in Figure 3, in general, both the SQ-2–biochar combination and the single application of SQ-2 enhanced the physicochemical properties of the rice rhizosphere soil. In the SQ-2–biochar group (S + M + C group), nitrate nitrogen, available potassium, and sucrase in the rhizosphere soil were significantly increased by 9.4%, 45.9%, and 466.8%, respectively, compared to those in the Al/Pb group (M) (Figure 3). It should be noted that the detection of sucrase utilized soil fresh weight, and in enzyme activity-related tests, fresh soil enzymes often exhibited higher activity than those in air-dried samples. The ammonium nitrogen in the soil of the SQ-2–biochar group (S + M + C) was found to be 22.6% higher than that in the Al/Pb group (M), although this increase was not statistically significant (Figure 3D). Furthermore, the SQ-2–biochar group (S + M + C group) demonstrated a significantly higher pH level compared to the other three groups (Figure 3E). However, the available phosphorus in the soil of the SQ-2–biochar group (S + M + C group) decreased by 15% compared to that in the Al/Pb group (M).

#### 3.3.3. Characterization of Pb^2+^ and Al^3+^ Adsorption Capacity by SQ-2

To elucidate the mechanism of Pb^2+^ and Al^3+^ adsorption by SQ-2, an analysis of the adsorption products was conducted using SEM-EDS, FT-IR, XRD, and XPS.

**Figure 4:**
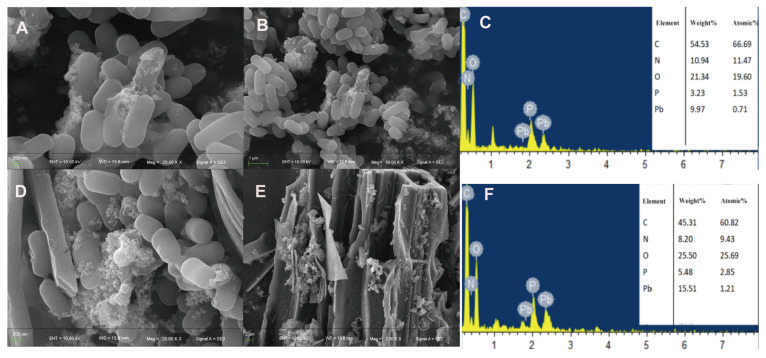
The adsorption effect of strain SQ-2 and SQ-2–biochar on Pb^2+^ according to scanning electron microscopy. (**A**): SEM image of Pb^2+^ adsorption by SQ-2 (200 nm); (**B**): SEM image of Pb^2+^ adsorption by SQ-2 (1 μm); (**C**): EDS spectra analysis of Pb^2+^ adsorption by SQ-2; (**D**): SEM image of Pb^2+^ adsorption by SQ-2–biochar (200 nm); (**E**): SEM image of Pb^2+^ adsorption by SQ-2–biochar (2 μm); (**F**): EDS spectra of Pb^2+^ adsorption by SQ-2–biochar.

We used SEM-EDS to observe the microstructure of bacterial cells following metal treatment and to determine heavy metal adsorption on the surfaces of bacteria and biochar. After exposing the strain to Pb^2+^ (Figure 4A,B), the bacteria exhibited short and thick rod-shaped structures with a relatively smooth surface. However, some cells adhered to each other, and a substantial number of granular chelates were attached to the cell surface. We hypothesized that Pb^2+^ precipitated and adsorbed onto the bacterial surface. Similarly, when the strain was treated with Pb^2+^ and biochar (Figure 4D,E), a large amount of Pb precipitate adsorbed on the surface of bacteria and biochar could be observed. But the amount of precipitate on the cell surface increased. And a large number of bacteria were observed adhering to the biochar.

**Figure 5:**
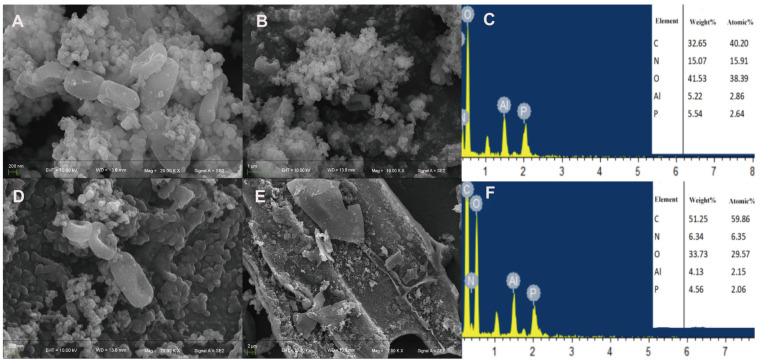
The adsorption effect of strain SQ-2 and SQ-2–biochar on Al^3+^ by scanning electron microscopy. (**A**): SEM image of Al^3+^ adsorption by SQ-2 (200 nm); (**B**): SEM image of Al^3+^ adsorption by SQ-2 (1 μm); (**C**): EDS spectra of Al^3+^ adsorption by SQ-2; (**D**): SEM image of Al^3+^ adsorption by SQ-2 -biochar (200 nm); (**E**): SEM image of Al^3+^ adsorption by SQ-2–biochar (2 μm); (**F**): EDS spectra of Al^3+^ adsorption by SQ-2–biochar.

After treating the strain with Al^3+^ (Figure 5A,B), a significant amount of spherical precipitates adhered to the cell surface, and the bacterial body underwent a certain degree of deformation and depression. When the strain was treated with Al^3+^ and biochar (Figure 5D,E), a large amount of sediment was adsorbed on the surfaces of the bacteria and biochar. It is hypothesized that Al^3+^ can better adhere to the extracellular polysaccharides produced by the bacteria, resulting in the even distribution of the metal on the surfaces of the bacteria and biochar. EDS analysis revealed that the SQ-2–biochar group contained a large amount of carbon (C), nitrogen (N), and oxygen (O) elements, and the corresponding metal elements were characterized after adding different metals (Figure 4C,F and Figure 5C,F).

**Figure 6:**
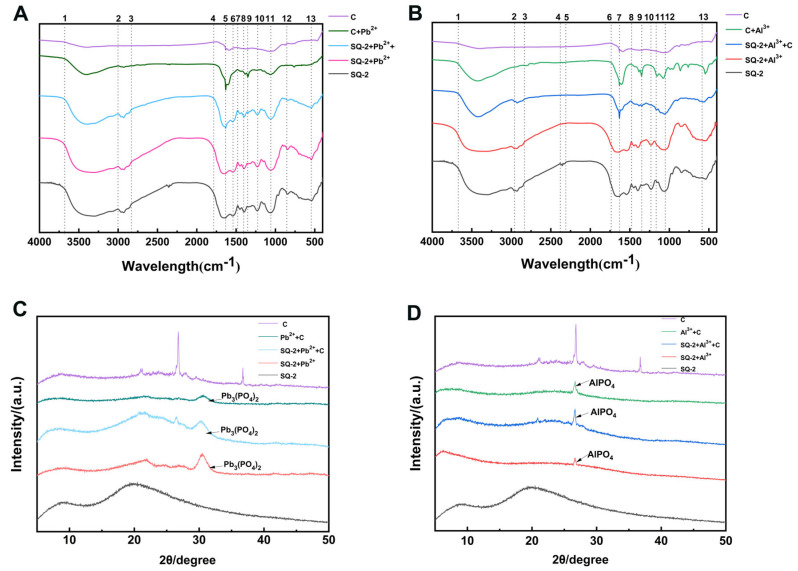
(**A**): FTIR spectrum of Pb^2+^ precipitation product. (Notes: 1. 3685.1 cm^−1^; 2. 3001.5 cm^−1^; 3. 2829.1 cm^−1^; 4. 1786.6 cm^−1^; 5. 1626.1 cm^−1^; 6. 1544.0 cm^−1^; 7. 1484.3 cm^−1^; 8. 1401.5 cm^−1^; 9. 1355.2 cm^−1^; 10. 1229.1 cm^−1^; 11. 1059.0 cm^−1^; 12. 849.3 cm^−1^; 13. 543.3 cm^−1^). (**B**): FTIR spectrum of Al^3+^ precipitation product. (Notes: 1. 3676.9 cm^−1^; 2. 2960.4 cm^−1^; 3. 2838.1 cm^−1^; 4. 2386.6 cm^−1^; 5. 2307.5 cm^−1^; 6. 1740.3 cm^−1^; 7. 1631.3 cm^−1^; 8. 1480.0 cm^−1^; 9. 1347.0 cm^−1^; 10. 1231.3 cm^−1^; 11. 1165.7 cm^−1^; 12. 1052.2 cm^−1^; 13. 578.6 cm^−1^). (**C**): XRD pattern of Pb^2+^ precipitation product. (**D**): XRD pattern of Al^3+^ precipitation product.

During the process of adsorption, metals have been observed to exert toxicity on bacteria, thereby prompting the activation of their defense mechanisms in order to resist external stimuli. Furthermore, the surface of bacteria is characterized by the presence of polysaccharides, proteins, and lipids, which possess a high adsorption capacity for metal ions. This property facilitates the mineralization and precipitation of metals, thereby reducing their toxicity [34]. In this study, we utilized FTIR to identify the functional groups involved in the adsorption process, thereby determining the extracellular reaction mechanism. Figure 6A,B display the FTIR spectra of Pb^2+^ and Al^3+^ adsorption by SQ-2–biochar, respectively.

As shown in Figure 6A, in the range of 3685.1–3001.5 cm^−1^ (notes 1–2), compared to the addition of biochar alone (C group), the C + Pb^2+^ group exhibited a strong and broad peak. Following the addition of the strain, an enhancement in peak intensity within this range was observed. It is presumed that there was an increase in the free -OH and -NH content of the substance or the presence of hydrogen bonding, which enhanced the vibration and absorption, resulting in broader infrared peaks. In the range of 3001.5–2829.1 cm^−1^ (notes 2–3), no significant peak was observed in the C group and C + Pb^2+^ group, while small peaks appeared in the other groups. This phenomenon is plausibly ascribed to the vibrations of -NH and -CH_2_ subsequent to the incorporation of the strain. All groups displayed a broad peak at 1786.6–1484.3 cm^−1^ (notes 4–7), with 1626.1 cm^−1^ (note 5) attributed to the C=O stretching vibration in the amide I band. The groups with added strains showed a peak at 1544.0 cm^−1^ (note 6), presumably N-H (amide II band). The groups with added strains also exhibited peak segments at 1229.1 and 1059.0 cm^−1^ (notes 10–11), indicating the stretching vibration of phosphate groups. The C + Pb^2+^ group had a peak at 1355.2 cm^−1^ (note 9), while the peaks of the three groups with added strains shifted, reaching a peak at 1401.5–1000 cm^−1^ (note 8). It is speculated that C-O, C-O-C, C-C, and C-O-P are also involved in the metal adsorption process [35].

As demonstrated in Figure 6B, within the range of 3676.9–2960.4 cm^−1^ (refer to notes 1–2), in comparison to the incorporation of biochar alone (C group), the stretching vibration of the C + Al^3+^ group and the SQ-2 + C + Al^3+^ group underwent alterations, whilst the peaks of the SQ-2 + Al^3+^ group and the SQ-2 group exhibited greater breadth than that observed for the aforementioned two groups. It is hypothesized that -NH and -OH on the bacterial surface participate in the adsorption process of metals, which is similar to the results of the Pb^2+^ group. In the range of 1740.3–1480.0 cm^−1^ (notes 7–9), all five groups exhibited C=O peaks in the amide I band and N-H peaks in the amide II band, with varying degrees of stretching vibration, indicating the involvement of these amide-related functional groups in the adsorption process. The SQ-2 + Al^3+^ group exhibited clear peaks at 1231.3 cm^−1^ and 1052.2 cm^−1^ (notes 10, 12), which are speculated to be the asymmetric and symmetric absorption bands of the phosphate diester group, suggesting the participation of the phosphate group in the adsorption of Al^3+^. The stretching vibration of -SO_3_ occurred between 1165.7 and 1000 cm^−1^ (note 11), which is considered to be a thioester bond or a thioketone bond. Peaks at 1400 cm^−1^ were observed in all groups containing strains, indicating the involvement of extracellular polymers in adsorption [36].

As demonstrated by the FTIR spectra, the surface of SQ-2 is characterized by the presence of a number of key functional groups, including C-O-C, C=O, N-H, P-O, and -O-H, which are believed to play a pivotal role in the process of adsorption for both Pb^2+^ and Al^3+^ ions.

An analysis was conducted of the physical characteristics of Pb^2+^ and Al^3+^ mineralized by SQ-2, with the use of XRD. As shown in Figure 6C, the Pb^2+^ group detected peak 2θ values at 21.04°, 24.36°, 26.48°, and 30.47°, indicating that Pb precipitated as Pb_3_(PO_4_)_2_. Compared to those of Pb^2+^ + C, the characteristic peaks of SQ-2 + Pb^2+^ and SQ-2 + Pb^2+^ + C were sharper, suggesting that the addition of bacteria facilitated the deposition of Pb^2^ on the bacterial surface. As shown in Figure 6D, the Al^3+^ group detected peak 2θ values at 20.72° and 26.56°, indicating that Al^3+^ precipitated as AlPO_4_.

**Figure 7:**
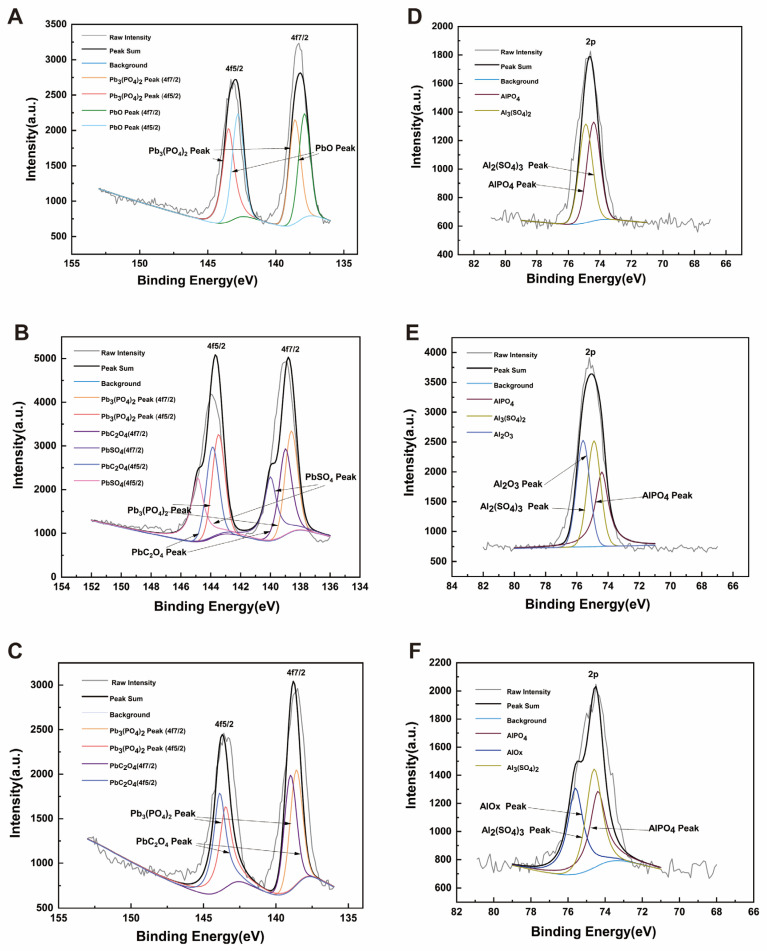
XPS spectrum of precipitation products. (**A**): SQ-2 + Pb^2+^; (**B**): biochar + Pb^2+^; (**C**): SQ-2–biochar + Pb^2+^; (**D**): SQ-2 + Al^3+^; (**E**): biochar + Al^3+^; (**F**): SQ-2–biochar + Al^3+^.

XPS was used to analyze the elemental composition, molecular structure, and atomic valence states of the compounds. As shown in Figure 7, Pb^2+^ was mainly precipitated as PbC_2_O_4_, PbSO_4_, and Pb_3_(PO_4_)_2_, while Al^3+^ was mainly precipitated as Al_2_(SO_4_)_3_ and AlPO_4_. The biochar used in the experiment was corn stover biochar, which may generate oxalate, sulfate, and phosphate. The precipitates of PbC_2_O_4_, PbSO_4_, and Al_2_(SO_4_)_3_ detected by XPS were likely formed by the combination of these substances with oxalate and sulfate in the biochar. SQ-2 is a biotrophic bacterium with a phosphorus-solubilizing function, capable of dissolving insoluble inorganic phosphorus into phosphate through acid production, thereby generating Pb_3_(PO_4_)_2_ and AlPO_4_ precipitates. Based on the results of XRD and XPS, it can be concluded that under the action of SQ-2 and biochar, Pb^2+^ can be converted into PbC_2_O_4_, PbSO_4_, and Pb_3_(PO_4_)_2_ precipitates. Concurrently, Al^3+^ is shown to be transformed into Al_2_(SO_4_)_3_ and AlPO_4_ precipitates, thereby facilitating the mineralization of Pb^2+^ and Al^3+^ and concomitantly attenuating the stress exerted by metals on rice.

### 3.4. Effects of Pb^2+^ and Al^3+^ on SQ-2 Genome Expression

**Figure 8:**
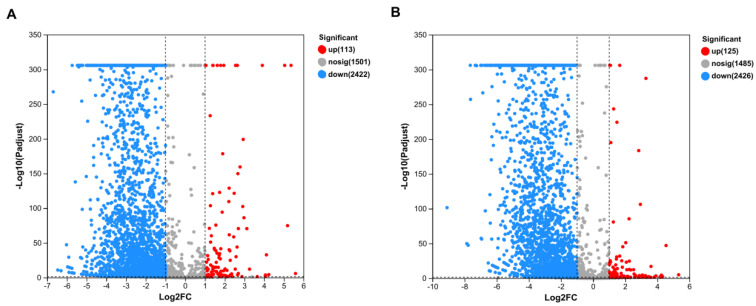
Transcriptome analysis of metal ion adsorption by strain SQ-2. (**A**): Volcano plot of SQ-2 under Pb^2+^ stress vs. CK. (**B**): Volcano plot of SQ-2 under Al^3+^ stress vs. CK.

To further clarify the mechanism of SQ-2 adsorption of Pb^2+^ and Al^3+^, we conducted transcriptome analysis. As shown in Figure 8A, a total of 2535 significantly differentially expressed genes were identified between the Pb^2+^ group and the CK group. Among them, 113 genes were found to be overexpressed, 2422 genes were found to be underexpressed, and 1502 genes remained unchanged. As shown in Figure 8B, a total of 2551 significantly differentially expressed genes were detected between the Al^3+^ group and the CK group. In this case, 125 genes were found to be overexpressed, 2426 genes were found to be underexpressed, and 1485 genes remained unchanged. A statistically significant disparity was observed in the number of downregulated genes, which significantly exceeded the number of overexpressed genes in both groups.

As presented in Table S1, the Pb group was found to have a higher number of upregulated DEGs (differentially expressed genes) compared to the CK (control) group, along with the names of some genes involved in KEGG pathways. Metal ABC transporters can bind to proteins and utilize the energy of ATP hydrolysis to transport various substances. In ligand/receptor interactions, it was found that the gene *znuA* related to metal ABC transporter binding proteins was overexpressed (2.9 times). In the pathway of synthesizing sensing proteins, the serine protease *thrB* gene was overexpressed (2.2 times). In the metabolic pathways of arginine and proline, the glutamate 5-phosphate kinase gene *proB* was overexpressed (2.4 times), and glutamate 5-phosphate kinase is the first step in proline synthesis. Mutations or deletions in the *proB* gene have been linked to disorders in proline synthesis.

Table S2 shows the annotation of up- and downregulated DEGs in the Al group versus the CK transcriptome and the names of some genes involved in KEGG pathways. In the metal ABC transport pathway, the upregulation of the genes *ssuC* (1.76 times), *opuCD* (1.28 times), *msmG* (1.90 times), *msmE* (1.73 times), *tcyK* (2.21 times), *nisG* (1.67 times) was observed.

## 4. Discussion

Based on the previously screened growth-promoting bacterium SQ-2, this study investigated the characteristics and mechanisms of SQ-2–biochar in promoting rice growth in Al/Pb-contaminated acidic soil. Specifically, the SQ-2–biochar group demonstrated increases of 41.9% in stem fresh weight, 33.3% in stem dry weight, 28.6% in root dry weight, 27.7% in root fresh weight, and 21.3% in plant height. Similarly, Nafees [31] et al. demonstrated that the co-application of biochar and PGPR resulted in a 30.4–180.4% increase in the leaf area index in leguminous plants. Hafez [37] et al. reported that biochar–PGPR co-amendment significantly enhanced grain and straw yields in rice. Collectively, these findings underscore the promising efficacy of biochar–PGPR co-application as a viable strategy for plant growth promotion.

Elements such as nitrogen, phosphorus, and potassium are essential for plant growth and development [38]. Nitrogen plays a crucial role in influencing organic structure, physiological properties, and biomass synthesis and distribution in plants [39]. Insufficient nitrogen supply severely impairs the structure and function of photosynthesis, thereby reducing crop yields [40]. In our research, SQ-2–biochar increased the soil nitrate and ammonium nitrogen contents by 9.4% and 23.1%, respectively. The relatively low availability of potassium and phosphorus in soil has been identified as a key factor in limiting crop yields. However, the presence of certain bacteria has been shown to facilitate the dissolution of these elements from soil. Specifically, phosphate-solubilizing rhizobacteria (PSB) have been observed to dissolve insoluble phosphorus in soil and produce organic acids [41]. Furthermore, certain rhizosphere bacteria, including *Pseudomonas*, *Bacillus*, and *Klebsiella*, have been shown to be capable of releasing potassium from insoluble minerals such as mica and illite [42]. In the present study, SQ-2 exhibited the ability to dissolve phosphorus and could form a phosphorus-solubilizing zone on PKO solid medium. The amount of dissolved phosphorus reached 245 mg/L on the sixth day. Phosphorus is an essential component of nucleic acids, membrane lipids, energy metabolites, and the activation of intermediates of the photosynthetic carbon cycle [43]. Additionally, inorganic phosphate is crucial in signal transduction cascades. Phosphorus deficiency in plants leads to a reduction in leaf area, alterations in photosynthesis and carbon metabolism, and ultimately, a decrease in rice yields [44]. SQ-2 also demonstrated the capacity to dissolve potassium, as the strain could adhere to the surface of potassium feldspar, causing it to become rough due to corrosion. Potassium is vital for plant metabolism, such as cell synthesis, enzyme activity, and protein and vitamin production. Furthermore, potassium has been shown to enhance the resistance of plants to abiotic and biotic stresses, as well as to regulate metabolic pathways [45]. Conversely, potassium deficiency has been demonstrated to result in slow plant growth and ultimately lower yields [46]. Among all macronutrients, potassium uptake is relatively higher, which is beneficial for the better growth and development of rice [47].

It has been reported that the exposure of plant cells to metals results in the generation of reactive oxygen species (ROS), leading to oxidative stress and affecting membrane permeability, photosynthetic reactions, and related enzyme activities [48]. PGPR has been shown to enhance ROS scavenging by upregulating genes involved in antioxidant enzyme synthesis [49]. Catalase and superoxide dismutase have been identified as pivotal in plant responses to oxidative stress by scavenging free radicals and mitigating oxidative damage, thereby ensuring the maintenance of cellular physiological homeostasis [50]. Plant phenolics, non-enzymatic antioxidants, have been shown to form metal chelates and participate in ROS scavenging [51]. The accumulation of malondialdehyde has been demonstrated to disrupt cell membranes and cellular metabolism, including photosynthesis and respiration [52]. In this study, catalase activity, superoxide dismutase activity, total plant phenol content, and malondialdehyde content were monitored as indicators of antioxidant capacity to elucidate the growth-promoting mechanism of PGPR. Specifically, catalase activity and superoxide dismutase activity increased by 162.5% and 162.9%, respectively. Concurrently, the total phenolic content exhibited a 17.6% increase, while the malondialdehyde content experienced a substantial decrease of 83.7%. These observations suggest that SQ-2–biochar plays a role in mitigating oxidative stress in rice. Additionally, it is well-documented that reduced chlorophyll content in rice under conditions of metal stress imposes limitations on photosynthesis, affects carbon fixation, and consequently reduces the growth capacity of the plant. Therefore, chlorophyll content serves as a crucial indicator of plant stress tolerance and productivity [53]. In the present study, the chlorophyll content exhibited an increase of 83.7%, signifying that SQ-2–biochar possesses the capacity to restore chlorophyll content and enhance photosynthesis and stress tolerance in rice under conditions of metal stress.

The combined application of biochar and PGPR has been demonstrated to enhance soil nutrients [33]. Soil sucrase, which is closely related to soil organic matter metabolism and nitrogen and phosphorus content, has been shown to characterize the level of soil fertility and soil biological activity. The enzyme reaction products have been demonstrated to directly affect the growth of crops [54]. In the present study, there was a 466.8% increase in soil sucrase activity, which may be attributed to SQ-2–biochar promoting the secretion of enzymes from rice roots into the soil, thereby enhancing soil enzyme activity. In addition, the ability of biochar to increase soil sucrase activity may be due to its protective effect on soil enzymes [55]. SQ-2–biochar increased the content of available potassium in the soil by 45.9%. This is attributed to the potassium-solubilizing ability of SQ-2 and the high potassium content of biochar. The soil pH increased to 6.1, which is related to the alkaline nature of biochar.

In addition, an investigation was conducted into the mechanism of SQ-2–biochar in removing Pb^2+^ and Al^3+^. Through SEM-EDS, XRD, XPS and FT-IR analyses were used to determine the removal process of Pb^2+^ and Al^3+^ ions by SQ-2–biochar, the result of which indicated that the removal was achieved through bioreduction, biosorption, and biomineralization. The precipitation mainly consisted of Pb_3_(PO_4_)_2_ and AlPO_4_. The adsorption process of Pb^2+^ and Al^3+^ by SQ-2–biochar was facilitated by functional groups such as C-O-C, C=O, N-H, P-O, and -O-H present on the surface of microorganisms. The mechanism of SQ-2 adsorbing Pb^2+^ and Al^3+^ was further elucidated through transcriptome analysis. Transcriptome studies have shown that under Pb^2+^ and Al^3+^ stress, bacteria have 94.8–95.3% more downregulated genes than upregulated genes. Similarly, Cao [56] et al. revealed that copper (Cu^2+^) stress drastically downregulates the transcription and expression of electron transfer chain-related genes involved in the TCA cycle and organic halide respiration processes. These findings collectively demonstrate the significant inhibitory effects of heavy metals on bacterial metabolic activities. Additionally, in both Pb^2+^ and Al^3+^ stress groups, there was no significant upregulation of pathways, and the common upregulated genes in both groups were concentrated in pathways related to metal ABC transport, sensing proteins, and cell wall formation. These changes may occur because bacteria can reduce metal toxicity by regulating the expression of relevant genes mediated to excrete heavy metals out of the cell and reduce the intracellular concentration of heavy metals.

## 5. Conclusions

In conclusion, the present study demonstrated that SQ-2–biochar was capable of promoting rice growth in Al/Pb-contaminated acidic soil. SQ-2–biochar has the ability to adsorb and mineralize Pb^2+^ and Al^3+^ ions, thereby alleviating the toxicity of metals on rice. Furthermore, SQ-2–biochar enhances soil fertility and improves the physiological properties of rice, thereby increasing its antioxidant capacity, photosynthesis, and stress tolerance. This study demonstrates that SQ-2–biochar has a favorable effect on the growth of rice in metal-contaminated acidic soils. The present study employed a series of pot experiments, which were designed to emulate the conditions prevalent during agricultural planting operations within a laboratory setting. This scientific investigation has yielded valuable theoretical insights and has established a robust empirical foundation for comprehending the collaborative promotion of plant growth by microorganisms and biochar under conditions of metal stress. Based on this study’s conclusion, subsequent work will investigate the efficacy of combined biochar–PGPR amendments across diverse plant species and advance their deployment in sustainable agricultural practices.

## Figures and Tables

**Table 1:** SQ-2–biochar promotes rice growth in Pb/Al soil.

Treatment	Root Dry Weight (g)	Stem Dry Weight (g)	Root Fresh Weight (g)	Stem Fresh Weight (g)	Stem Thickness (mm)	Plant Height (cm)
CK	0.101 ± 0.003 a	0.051 ± 0.002 a	1.022 ± 0.014 a	0.48 ± 0.02 a	0.78 ± 0.01 a	12.3 ± 0.2 a
M	0.072 ± 0.002 c	0.032 ± 0.001 b	0.651 ± 0.015 c	0.31 ± 0.01 b	0.72 ± 0.02 b	9.7 ± 0.2 c
S + M	0.082 ± 0.002 bc	0.041 ± 0.003 a	0.773 ± 0.018 bc	0.43 ± 0.03 a	0.74 ± 0.01 ab	11.5 ± 0.2 b
S + M + C	0.091 ± 0.001 bc	0.042 ± 0.002 a	0.831 ± 0.012 b	0.44 ± 0.01 a	0.74 ± 0.02 ab	11.7 ± 0.2 ab

Units: CK: −Al/Pb, −*B. amyloliquefaciens* SQ-2, –biochar; M: +Al/Pb, −*B. amyloliquefaciens* SQ-2, –biochar; S + M: +Al/Pb, +*B. amyloliquefaciens* SQ-2, –biochar; S + M + C: +Al/Pb, +*B. amyloliquefaciens* SQ-2, +biochar. Data are expressed as the mean ± standard deviation (n = 3). Different letters in the same column indicate significant differences at the *p* < 0.05 level.

## Data Availability

The original contributions presented in this study are included in the article. Further inquiries can be directed to the corresponding authors.

## Supplementary Materials

The following supporting information can be downloaded at: https://www.mdpi.com/article/10.3390/microorganisms13071556/s1, Figure S1: The effect of SQ-2-biochar on seeding stage growth of rice in Al/Pb soil.; Table S1: Annotations of upregulated and downregulated DEGs in the Pb group vs CK transcriptome and partial gene names involved in KEGG; Table S2: Annotations of upregulated and downregulated DEGs in the Al group vs CK transcriptome and partial gene names involved in KEGG.

## References

[1] Xiang M., Li Y., Yang J., Lei K., Li Y., Li F., Zheng D., Fang X., Cao Y. (2021). Metal contamination risk assessment and correlation analysis of metal contents in soil and crops. Environ. Pollut..

[2] Fasahat P. (2015). Recent progress in understanding cadmium toxicity and tolerance in rice. Emir. J. Food Agric..

[3] Meena R.S., Kumar S., Bohra J.S., Lal R., Yadav G.S., Pandey A. (2019). Response of alley cropping-grown sesame to lime and sulphur on yield and available nutrient status in an acidic soil of Eastern India. Energy Ecol. Environ..

[4] Shetty R., Prakash N.B. (2020). Effect of different biochars on acid soil and growth parameters of rice plants under aluminium toxicity. Sci. Rep..

[5] Rigoletto M., Calza P., Gaggero E., Malandrino M., Fabbri D. (2020). Bioremediation methods for the recovery of lead-contaminated soils: A review. Appl. Sci..

[6] Sun Y., Li Y., Xu Y., Liang X., Wang L. (2015). In situ stabilization remediation of cadmium (Cd) and lead (Pb) co-contaminated paddy soil using bentonite. Appl. Clay Sci..

[7] Lin X.-R., Chen H.-B., Li Y.-X., Zhou Z.-H., Li J.-B., Wang Y.-Q., Zhang H., Zhang Y., Han Y.-H., Wang S.-S. (2022). *Priestia* sp. LWS1 Is a Selenium-Resistant Plant Growth-Promoting Bacterium That Can Enhance Plant Growth and Selenium Accumulation in *Oryza sativa* L.. Agronomy.

[8] Khumairah F.H., Setiawati M.R., Fitriatin B.N., Simarmata T., Alfaraj S., Ansari M.J., El Enshasy H.A., Sayyed R.Z., Najafi S. (2022). Halotolerant plant growth promoting rhizobacteria isolated from saline soil improve nitrogen fixation and alleviate salt stress in rice plants. Front. Microbiol..

[9] Bach E., Lisboa B.B., Passaglia L.M.P. (2016). Evaluation of biological control and rhizosphere competence of plant growth promoting bacteria. Appl. Soil Ecol..

[10] Ahemad M. (2012). Implications of bacterial resistance against metals in bioremediation: A review. J. Inst. Integr. Omics Appl. Biotechnol. (IIOAB).

[11] Akinrinlola R.J., Yuen G.Y., Drijber R.A., Adesemoye A.O. (2018). Evaluation of Bacillus strains for plant growth promotion and predictability of efficacy by in vitro physiological traits. Int. J. Microbiol..

[12] Cui W., He P., Munir S., He P., Li X., Li Y., Wu J., Wu Y., Yang L., He P. (2019). Efficacy of plant growth promoting bacteria *Bacillus amyloliquefaciens* B9601-Y2 for biocontrol of southern corn leaf blight. Biol. Control..

[13] Egamberdieva D., Adesemoye A.O. (2016). Improvement of crop protection and yield in hostile agroecological conditions with PGPR-based biofertilizer formulations. Bioformulations: For Sustainable Agriculture.

[14] Govindasamy V., Senthilkumar M., Magheshwaran V., Kumar U., Bose P., Sharma V., Annapurna K. (2011). *Bacillus* and *Paenibacillus* spp.: Potential PGPR for sustainable agriculture. Plant Growth and Health Promoting Bacteria.

[15] Ongena M., Jacques P. (2008). *Bacillus lipopeptides*: Versatile weapons for plant disease biocontrol. Trends Microbiol..

[16] Kavitha B., Reddy P.V.L., Kim B., Lee S.S., Pandey S.K., Kim K.-H. (2018). Benefits and limitations of biochar amendment in agricultural soils: A review. J. Environ. Manag..

[17] Qayyum M.F., Haider G., Raza M.A., Mohamed A.K.S., Rizwan M., El-Sheikh M.A., Alyemeni M.N., Ali S. (2020). Straw-based biochar mediated potassium availability and increased growth and yield of cotton (*Gossypium hirsutum* L.). J. Saudi Chem. Soc..

[18] Alvarez-Campos O., Lang T.A., Bhadha J.H., McCray J.M., Glaz B., Daroub S.H. (2018). Biochar and mill ash improve yields of sugarcane on a sand soil in Florida. Agric. Ecosyst. Environ..

[19] Mehmood A., Hussain A., Irshad M., Hamayun M., Iqbal A., Khan N. (2019). In vitro production of IAA by endophytic fungus *Aspergillus awamori* and its growth promoting activities in *Zea mays*. Symbiosis.

[20] Batool M., Khan W.-U., Hamid Y., Farooq M.A., Naeem M.A., Nadeem F. (2022). Interaction of pristine and mineral engineered biochar with microbial community in attenuating the heavy metals toxicity: A Review. Appl. Soil Ecol..

[21] Wu C., Zhi D., Yao B., Zhou Y., Yang Y., Zhou Y. (2022). Immobilization of microbes on biochar for water and soil remediation: A Review. Environ. Res..

[22] Bandara T., Franks A., Xu J., Bolan N., Wang H., Tang C. (2020). Chemical and biological immobilization mechanisms of potentially toxic elements in biochar-amended soils. Crit. Rev. Environ. Sci. Technol..

[23] Tu C., Wei J., Guan F., Liu Y., Sun Y., Luo Y. (2020). Biochar and bacteria inoculated biochar enhanced Cd and Cu immobilization and enzymatic activity in a polluted soil. Environ. Int..

[24] Zhao S., Zhao S., Wang B. (2025). Combined application of biochar and PGPB on crop growth and heavy metals accumulation: A meta-Analysis. Environ. Pollut..

[25] Li S., Dai S., Huang L., Cui Y., Ying M. (2024). Biocontrol Ability of Strain *Bacillus amyloliquefaciens* SQ-2 against Table Grape Rot Caused by *Aspergillus tubingensis*. J. Agric. Food Chem..

[26] Zak D., Goldhammer T., Cabezas A., Gelbrecht J., Gurke R., Wagner C., Reuter H., Augustin J., Klimkowska A., McInnes R. (2018). Top soil removal reduces water pollution from phosphorus and dissolved organic matter and lowers methane emissions from rewetted peatlands. J. Appl. Ecol..

[27] Liu T., Xu T., Yu F., Yuan Q., Guo Z., Xu B. (2021). A method combining ELM and PLSR (ELM-P) for estimating chlorophyll content in rice with feature bands extracted by an improved ant colony optimization algorithm. Comput. Electron. Agric..

[28] Quiles F.A., Galvez-Valdivieso G., Guerrero-Casado J., Pineda M., Piedras P. (2019). Relationship between ureidic/amidic metabolism and antioxidant enzymatic activities in legume seedlings. Plant Physiol. Biochem..

[29] Sanchez-Gonzalez M.E., Mora-Herrera M.E., Wong-Villarreal A., De La Portilla-López N., Sanchez-Paz L., Lugo J., Vaca-Paulín R., Del Aguila P., Yañez-Ocampo G. (2022). Effect of pH and carbon source on phosphate solubilization by bacterial strains in pikovskaya medium. Microorganisms.

[30] Flores J.P.M., Alves L.A., de Oliveira Denardin L.G., Martins A.P., Bortoluzzi E.C., Inda A.V., de Faccio Carvalho P.C., Tiecher T. (2021). Soil K forms and K budget in integrated crop-livestock systems in subtropical paddy fields. Soil Tillage Res..

[31] Nafees M., Ullah S., Ahmed I. (2022). Modulation of drought adversities in Vicia faba by the application of plant growth promoting rhizobacteria and biochar. Microsc. Res. Tech..

[32] Wang D., Gao Y., Sun S., Lu X., Li Q., Li L., Wang K., Liu J. (2022). Effects of salt stress on the antioxidant activity and malondialdehyde, solution protein, proline, and chlorophyll contents of three Malus species. Life.

[33] Malik L., Sanaullah M., Mahmood F., Hussain S., Shahzad T. (2024). Co-application of biochar and salt tolerant PGPR to improve soil quality and wheat production in a naturally saline soil. Rhizosphere.

[34] Deng S., Dong H., Lv G., Jiang H., Yu B., Bishop M.E. (2010). Microbial dolomite precipitation using sulfate reducing and halophilic bacteria: Results from Qinghai Lake, Tibetan Plateau, NW China. Chem. Geol..

[35] D’Souza L., Devi P., Divya Shridhar M.P., Naik C.G. (2008). Use of Fourier Transform Infrared(FTIR)Spectroscopy to Study Cadmium-Induced Changes in *Padina tetrastromatica* (Hauck). Anal. Chem. Insights.

[36] Naik M.M., Pandey A., Dubey S.K. (2012). Biological characterization of lead-enhanced exopolysaccharide produced by a lead resistant Enterobacter cloacae strain P2B. Biodegradation.

[37] Hafez E.M., Alsohim A.S., Farig M., Omara A.E.-D., Rashwan E., Kamara M.M. (2019). Synergistic effect of biochar and plant growth promoting rhizobacteria on alleviation of water deficit in rice plants under salt-affected soil. Agronomy.

[38] Ye T., Li Y., Zhang J., Hou W., Zhou W., Lu J., Xing Y., Li X. (2019). Nitrogen, phosphorus, and potassium fertilization affects the flowering time of rice (*Oryza sativa* L.). Glob. Ecol. Conserv..

[39] Schmierer M., Knopf O., Asch F. (2021). Growth and photosynthesis responses of a super dwarf rice genotype to shade and nitrogen supply. Rice Sci..

[40] Zhao L.-S., Li K., Wang Q.-M., Song X.-Y., Su H.-N., Xie B.-B., Zhang X.-Y., Huang F., Chen X.-L., Zhou B.-C. (2017). Nitrogen starvation impacts the photosynthetic performance of *Porphyridium cruentum* as revealed by chlorophyll a fluorescence. Sci. Rep..

[41] Bashir Z., Zargar M.Y., Vishwakarma D.K. (2019). Potassium-solubilizing microorganisms for sustainable agriculture. Appl. Agric. Pract. Mitigating Clim. Change.

[42] Meena V.S., Maurya B.R., Verma J.P., Aeron A., Kumar A., Kim K., Bajpai V.K. (2015). Potassium solubilizing rhizobacteria (KSR): Isolation, identification, and K-release dynamics from waste mica. Ecol. Eng..

[43] Wang Y., Chen Y.F., Wu W.H. (2021). Potassium and phosphorus transport and signaling in plants. J. Integr. Plant Biol..

[44] Xu H.X., Weng X.Y., Yang Y. (2007). Effect of phosphorus deficiency on the photosynthetic characteristics of rice plants. Russ. J. Plant Physiol..

[45] Maqsood M., Shehzad M.A., Wahid A., Butt A.A. (2013). Improving Drought Tolerance in Maize (*Zea mays*) with Potassium Application in Furrow Irrigation Systems. Int. J. Agric. Biol..

[46] Gupta G., Parihar S.S., Ahirwar N.K., Snehi S.K., Singh V. (2015). Plant growth promoting rhizobacteria (PGPR): Current and future prospects for development of sustainable agriculture. J. Microb. Biochem. Technol..

[47] Fageria N.K. (2015). Potassium requirements of lowland rice. Commun. Soil Sci. Plant Anal..

[48] Geng A., Wang X., Wu L., Wang F., Wu Z., Yang H., Chen Y., Wen D., Liu X. (2018). Silicon improves growth and alleviates oxidative stress in rice seedlings (*Oryza sativa* L.) by strengthening antioxidant defense and enhancing protein metabolism under arsanilic acid exposure. Ecotoxicol. Environ. Saf..

[49] Feng L., Li Q., Zhou D., Jia M., Liu Z., Hou Z., Ren Q., Ji S., Sang S., Lu S. (2024). *B. subtilis* CNBG-PGPR-1 induces methionine to regulate ethylene pathway and ROS scavenging for improving salt tolerance of tomato. Plant J..

[50] Ren X., Zhu J., Liu H., Xu X., Liang C. (2018). Response of antioxidative system in rice (*Oryza sativa*) leaves to simulated acid rain stress. Ecotoxicol. Environ. Saf..

[51] Bhadwal S., Sharma S., Singh D. (2024). Interactive effects of selenium and arsenic on phenolic constituents and antioxidant activity in rice (*Oryza sativa* L.). Chemosphere.

[52] Kaya C., Ashraf M., Alyemeni M.N., Rinklebe J., Ahmad P. (2023). Citric acid and hydrogen sulfide cooperate to mitigate chromium stress in tomato plants by modulating the ascorbate-glutathione cycle, chromium sequestration, and subcellular allocation of chromium. Environ. Pollut..

[53] Zhi Y., Li X., Wang X., Jia M., Wang Z. (2024). Photosynthesis promotion mechanisms of artificial humic acid depend on plant types: A hydroponic study on C3 and C4 plants. Sci. Total Environ..

[54] Rudinskienė A., Marcinkevičienė A., Velička R., Kosteckas R., Kriaučiūnienė Z., Vaisvalavičius R. (2022). The comparison of soil agrochemical and biological properties in the multi-cropping farming systems. Plants.

[55] Oleszczuk P., Jośko I., Futa B., Pasieczna-Patkowska S., Pałys E., Kraska P. (2014). Effect of pesticides on microorganisms, enzymatic activity and plant in biochar-amended soil. Geoderma.

[56] Cao D., Chen X., Nan J., Wang A., Li Z. (2023). Biomolecular insights into the inhibition of heavy metals on reductive dechlorination of 2,4,6-trichlorophenol in *Pseudomonas* sp. CP-1. Water Res..

