# Supplementary material for: Bacillus amyloliquefaciens SQ-2 and Biochar: A Promising Combination for Enhancing Rice Growth in Pb/Al-Contaminated Acidic Soils
Source: Microorganisms. 2025 Jul 2;13(7):1556. doi: 10.3390/microorganisms13071556 (PMC12298256; doi:10.3390/microorganisms13071556)
Supplement: Supplementary file 1 [file microorganisms-13-01556-s001.zip › microorganisms-3711535-supplementary.pdf]

List of Figures and tables

Fig. S1. The effect of SQ-2-biochar on seeding stage growth of rice in Al/Pb soil.

Table S1. Annotations of upregulated and downregulated DEGs in the Pb group vs CK transcriptome and partial gene names involved in KEGG.

Table S2. Annotations of upregulated and downregulated DEGs in the Al group vs CK transcriptome and partial gene names involved in KEGG.

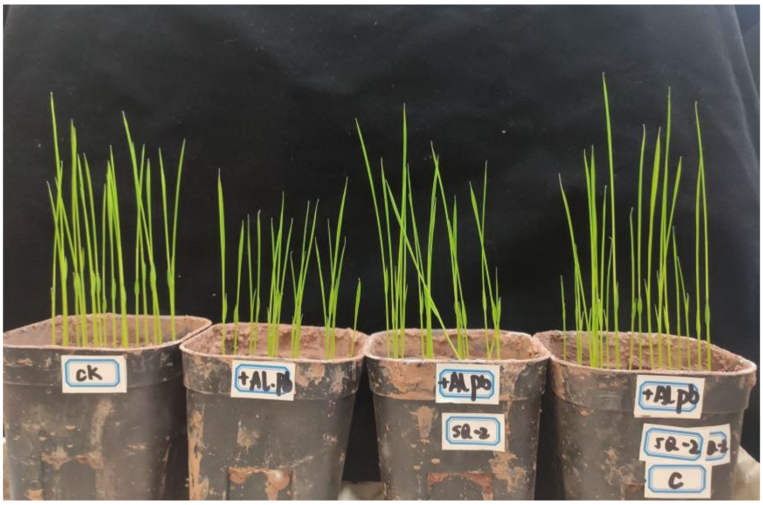

Fig S1. The effect of SQ-2-biochar on seeding stage growth of rice in Al/Pb soil.

TABLE S1 Annotations of upregulated and downregulated DEGs in the Pb group vs CK transcriptome and partial gene names involved in KEGG

Gene name	Gene description	log2FC	Up/Down
KZ489_RS02775	YoqO family proteins (membrane components)	5.58	Up
KZ489_RS04705	CsbA family proteins (membrane components)	5.35	Up
KZ489_RS13505(*coxA*)	YhcN/YlaJ family spore-producing lipoproteins	4.23	Up
KZ489_RS07670(*sda*)	sporulation histidine kinase inhibitor Sda	4.06	Up
KZ489_RS03070	D-Ala-phosphobiotic acid biosynthetic protein	4.06	Up
KZ489_RS02330(*spollSA*)	Antitoxin system (SpoIISA toxin)	3.11	Up
KZ489_RS18045(*rplGB*)	50S ribosomal protein l7ae-like protein	2.93	Up
KZ489_RS20320	NfeD family proteins (membrane components)	2.91	Up
KZ489_RS05005(*paiA*)	GNAT family (N-acetyltransferases)	2.86	Up
KZ489_RS14080	Proteins containing the DUF378 structural domain	2.77	Up
KZ489_RS11875	Amino acid proteins (PTP); Transporter proteins	2.17	Up
KZ489_RS15295(*sspA*)	Serine protease	2.00	Up
KZ489_RS01210(*spoVD*)	Peptidoglycan biosynthesis	1.64	Up
KZ489_RS15080(*znuA*)	Metallic ABC transporter-binding protein	1.55	Up
KZ489_RS02200(*pro*B)	Glutamate 5-kinase	1.28	Up
KZ489_RS14525(*thr*B)	Serine kinase (SerKin), an amino acid	1.15	Up
KZ489_RS19090(*dexA*)	Glycoside hydrolase family (glycoside hydrolases)	1.13	Up
KZ489_RS01065(*pyrB*)	Aspartate carbamoyltransferase (map)	-6.70	Down

KZ489_RS00075	histidine kinase (HisK), an essential amino acid	-6.47	Down
KZ489_RS00720(*flgB*)	flagellar matrix proteins	-5.97	Down
KZ489_RS03530	Proteins containing the DUF1700 structural domain	-5.91	Down
KZ489_RS13895(*satA*)	Streptomycin N-acetyltransferase	-5.90	Down
KZ489_RS00255(*acpK*)	Acyl carrier protein	-5.90	Down
KZ489_RS19280(*hxlA*)	3-Hexoketose-6-phosphate synthase	-5.87	Down
KZ489_RS16290	EcsC family proteins	-5.75	Down
KZ489_RS01060(*pyrC*)	dihydrolactamase	-5.74	Down
KZ489_RS13845(*ybaK*)	Cys tRNA (Pro) deacetylase	-5.70	Down
KZ489_RS15675(*treP*)	PTS system alginate-specific proteins	-2.78	Down
KZ489_RS15710(*nagE*)	N-acetylglucosamine specific PTS transporter protein	-2.28	Down
KZ489_RS01835(*ptsG*)	Glucose-specific PTS transporter protein	-2.06	Down
KZ489_RS01825 (*ptsP*)	phosphotransporter protein	-1.56	Down

TABLE S2. Annotations of upregulated and downregulated DEGs in the Al group vs CK transcriptome and partial gene names involved in KEGG

Gene name	Gene description	log2FC	Up/Down
KZ489_RS13075	AMP-binding protein	5.31	Up
KZ489_RS02775	YoqO Family Proteins	4.31	Up
KZ489_RS18975	Ghitin	3.90	Up
KZ489_RS14855	Helix-turn-Helix structural domain proteins	3.90	Up
KZ489_RS09060	M20/M25/M40 series metal hydrolases	3.80	Up
KZ489_RS03120	YwbE Family Proteins	3.59	Up
KZ489_RS20115(*bofA*)	Pro-sigmaK-treated protein	3.58	Up
KZ489_RS10250	5'-3'-Deoxyribonucleotidase	3.45	Up
KZ489_RS03375(*rfbA*)	Glucosinolate	2.99	Up
KZ489_RS18175(*ctsR*)	Transcriptional regulator CtsR	2.87	Up
KZ489_RS05455(*tycK*)	Amino acid ABC transporter protein substrate binding protein	2.2	Up
KZ489_RS18580(*msmG*)	Carbohydrate ABC transporter protein transferase	1.90	Up
KZ489_RS09210(*ssuC*)	ABC transporter protease	1.75	Up
KZ489_RS18590(*msmE*)	Extracellular solute binding protein	1.73	Up
KZ489_RS13865(*manA*)	Mannose-6-phosphate isomerase	1.71	Up
KZ489_RS05370(*nisG*)	Antibiotic-immunized ABC transporter protein MutG family	1.67	Up
KZ489_RS15785(*srfAB*)	Proteins containing condensed structural domains	1.51	Up
KZ489_RS13000(*scoA*)	CoA transferase subunit A	1.49	Up
KZ489_RS01210(*spoVD*)	Peptidoglycan synthesis	1.37	Up
KZ489_RS08485(*atoB*)	Acetyl coenzyme A acyltransferase	1.29	Up

KZ489_RS05330(*opuCD*)	Glycine betaine/carnitine/choline/choline sulfate ABC Transporter protein transferase	1.28	Up
KZ489_RS17885(*rpsM*)	30S ribosomal protein S13	-7.87	Down
KZ489_RS03180(*slrA*)	SinI family proteins	-7.78	Down
KZ489_RS02320	DUF47 domain-containing protein	-7.65	Down
KZ489_RS01065(*pyrB*)	Aspartate carbamoyltransferase catalytic subunit	-7.63	Down
KZ489_RS06205(*folE*)	GTP cyclic hydrolase I	-7.34	Down
KZ489_RS14715(*glmS*)	6-Phosphate transaminase	-7.30	Down
KZ489_RS01015(*sat*)	Sulfate adenylate transferase	-7.26	Down
KZ489_RS11240(*ackA*)	Acetate kinase	-7.00	Down
KZ489_RS20285(*trkA*)	TrkA family potassium uptake proteins	-6.97	Down
